# DNA Methylation Signature of Epileptic Encephalopathy-Related Pathogenic Genes Encoding Ion Channels in Temporal Lobe Epilepsy

**DOI:** 10.3389/fneur.2021.692412

**Published:** 2021-07-29

**Authors:** Hua Tao, Zengqiang Chen, Jianhao Wu, Jun Chen, Yusen Chen, Jiawu Fu, Chaowen Sun, Haihong Zhou, Wangtao Zhong, Xu Zhou, Keshen Li

**Affiliations:** ^1^Department of Neurology, Affiliated Hospital of Guangdong Medical University, Zhanjiang, China; ^2^Guangdong Key Laboratory of Age-Related Cardiac and Cerebral Diseases, Affiliated Hospital of Guangdong Medical University, Zhanjiang, China; ^3^Neurology & Neurosurgery Division, Stroke Center, Clinical Medicine Research Institute & The First Affiliated Hospital, Jinan University, Guangzhou, China

**Keywords:** DNA methylation, epigenetic, temporal lobe epilepsy, genetic susceptibility, ion channels

## Abstract

Epilepsy is characterized by highly abnormal synchronous discharge of brain neurons, and ion channels are fundamental in the generation and modulation of neural excitability. Considering that abnormal methylation can either activate or repress genes, this study was designed to explore the DNA methylation signature of pathogenic genes encoding ion channels in temporal lobe epilepsy (TLE). In total, 38 TLE patients and 38 healthy controls were enrolled in the study, and genomic DNA and total protein of the lymphocytes were extracted from peripheral blood samples to assess methylation and protein levels. The DNA methylation levels of all 12 genes examined were significantly lower in the TLE group than in the control group. After false-positive correction, 83.3% (10/12) of these genes, namely, gamma-aminobutyric acid type A receptor subunit beta1 *(GABRB1)*, gamma-aminobutyric acid type A receptor subunit beta2 *(GABRB2)*, gamma-aminobutyric acid type A receptor subunit beta1 *(GABRB3)*, glutamate ionotropic receptor NMDA type subunit 1 *(GRIN1)*, glutamate ionotropic receptor NMDA type subunit 2A *(GRIN2A)*, glutamate ionotropic receptor NMDA type subunit 2B *(GRIN2B)*, hyperpolarization activated cyclic nucleotide gated potassium channel 1 *(HCN1)*, potassium voltage-gated channel subfamily A member 2 *(KCNA2)*, potassium voltage-gated channel subfamily B member 1 *(KCNB1)*, and potassium sodium-activated channel subfamily T member 1 (*KCNT1)*, were still differentially expressed. Among these ion channels, *HCN1* and *KCNA2* were selected to evaluate the effects of DNA methylation, and the levels of these proteins were inversely upregulated in the TLE group compared to the control group. As the genes identified as having differential methylation levels are involved in both excitatory and inhibitory ion channels, this study observed by binary logistic regression that hypermethylated *GARAB1* was an independent risk factor for TLE, indicating that the overwhelming effect of ion channels on TLE is probably inhibitory from the perspective of DNA methylation. All these findings support the involvement of DNA methylation in TLE pathologies, but the mechanisms need to be further investigated.

## Introduction

Temporal lobe epilepsy (TLE) is the most common form of human epilepsy and has a high chance of becoming medically intractable. Thus, great efforts have been made to investigate its etiologies and pathologies in attempts to develop reliable strategies for evaluating predisposition toward TLE as well as novel drugs for treatment. TLE is traditionally considered a heterogeneous syndrome resulting from multiple environmental factors, such as perinatal asphyxia and febrile seizures (1), but accumulating evidence in recent decades highlights the influence of single-nucleotide polymorphisms (SNPs) and rare mutations in exons (2). Notably, in past year years, emerging evidence has indicated that epigenetic changes to genomic DNA, such as methylation, regulate gene function via certain modifications to chromatin structure (3).

DNA methylation is a major epigenetic modification that adds a methyl group to the fifth carbon of cytosine to form 5-methylcytosine; when present within cytosine-phosphate-G (CpG) islands proximal to promoters, such modification downregulates genes via transcriptional silencing (4). As it can be inherited by cell division, DNA methylation plays a crucial role in genetic regulation, with important implications for normal biology and disease (5). In one study, methylation analysis of all CpG islands proximal to promoters of the human genome revealed 146 protein-coding genes with an altered DNA methylation status in TLE patients with hippocampal sclerosis compared to those without hippocampal sclerosis, with 81.5% of the promoters displaying hypermethylation (6). Moreover, the expression of DNA methyltransferase 1 and 3a, which facilitate DNA methylation, is increased in the brain tissues of TLE patients. In addition, the status of DNA methylation can predict the risk of drug resistance in TLE patients (7). All this evidence supports the involvement of DNA methylation in seizure susceptibility and maintenance of the disorder.

Epilepsy is characterized by highly abnormal synchronous discharge of brain neurons, and ion channels are fundamental to excitability generation and modulation (8). Most of the variations identified to date in epilepsy patients are also common SNPs in healthy individuals; thus, significant differences between them do not necessarily prove a causal relationship (9). As these variations may be located within flanking, intronic and intergenic regions, they are often considered genetic markers (10–12). In comparison, exon mutations often result in abnormalities in protein structures and function (13). Based on *de novo* variants of exons in epileptic encephalopathies and additional standards (recurrence of variants in unrelated patients, information on previously identified phenotypes, and data from genetic studies), 17 ion-channel genes are considered to be pathogenic or possibly pathogenic (14). Epileptic encephalopathies are a devastating group of severe epilepsy in infants and children, including Dravet syndrome and West syndrome, which often display some features of TLE in adults (15). Several pathogenic genes with *de novo* mutations, such as sodium voltage-gated channel alpha subunit 1 (*SCN1A)* (16), are also involved in TLE, suggesting that *de novo* variants identified in epileptic encephalopathies might function in TLE.

Overall, increasing evidence indicates that DNA methylation and *de novo* mutations in exons coexist in many diseases (17–19). Therefore, this study aimed to evaluate the DNA methylation signature of the 17 ion channel genes identified in epileptic encephalopathies via *de novo* mutations, with the goal of deeply exploring pathologies in TLE patients from an epigenetic perspective.

## Materials and Methods

### Human Participants

In total, 38 TLE patients and 38 healthy controls were enrolled from the Affiliated Hospital of Guangdong Medical University. All experimental protocols involving human subjects were approved by the Ethics Committees of the Affiliated Hospital of Guangdong Medical University (18B1018B501). All human participants were Han Chinese, and informed consent was obtained at the time of their enrollment. The diagnosis of TLE was made on the basis of a constellation of clinical, EEG, and MRI criteria, which mainly referred to typical temporal auras or interictal EEG discharges within temporal lobes. Any sign of seizure onset outside the temporal structures by semiology or EEG findings was an exclusion criterion. Before enrollment, healthy controls were free of seizures and any other symptoms involved in neurological disorders, and no disease was diagnosed under routine physical examination items. For the TLE group, age at onset, disease duration and drug response were recorded. According to the definition of drug-resistant epilepsy proposed in 2010 by the commission of the International League Against Epilepsy (20), the responses to anti-epileptic drugs of the TLE patients were classified as follows: patients with drug-resistant epilepsy were determined on the basis of observation, namely, the absence of significant change or reduction in seizure frequency (<60%) or even augmentation after 1-year of treatment, with a schedule of at least two tolerated and properly selected anti-epileptic drugs; the remaining patients were considered to have drug-sensitive epilepsy.

### Methylation Experiments

First, CpG islands adjacent to the promoter region (from −2 to 1 kb downstream of the first exon) of the 17 ion channel genes were evaluated based on the following criteria: observed/expected ratio >0.60; percent of alleles C+G > 50.00%; length >200 bp. PCR primers were designed to facilitate multiplexable amplification of bisulfate-converted DNA using Methylation Primer software (Tiangen Biotech, Beijing) and the sequence information for CpG islands (referring to *Homo sapiens* build CBI37/hg19), and a mixed panel of these primers and their concentrations were further optimized to meet the highest efficiency and specificity.

Peripheral blood samples (3 ml) were collected from each participant, after which genomic DNA was extracted using an automatic nucleic acid extractor (Tiangen Biotech, Beijing). The genomic DNA was subjected to bisulfite treatment using the EZ DNA Methylation-Gold kit following the manufacturer's protocol (Zymo Research, CA, USA) to transform unmethylated allele C to allele U. The bisulfite-treated genomic DNA was amplified using the optimized panel of primer mixtures and the HotStarTaq polymerase kit (TAKARA, Tokyo, Japan). The biotinylated primers were further used for library construction, and the biotinylated products were sequenced using the paired-end sequencing protocol (2^*^150 bp) in accordance with the manufacturer's protocol (Illumina HiSeq Benchtop Sequencer, CA, USA). After a series of quality controls and data analyses, the levels of CpG methylation were equal to the rate of the reads of C and C+U alleles (Genesky Biotechnologies Inc., Shanghai, China).

### Protein Detection

According to the manufacturer's protocol, lymphocytes were obtained from the peripheral blood (2 ml) of each participant using lymphocyte separation media (Beijing Solarbio Science & Technology Co., Ltd., Beijing, China). Total protein was extracted from lymphocytes, and the levels of ion channel proteins, hyperpolarization activated cyclic nucleotide gated potassium channel 1 (*HCN1*) and potassium voltage-gated channel subfamily A member 2 (*KCNA2*) were measured using enzyme-linked immunosorbent assay (ELISA) kits (R&D Systems, Minneapolis, USA) following the manufacturer's instructions. Absorbance was measured using an ELISA reader (Bio-Rad Laboratories, Hercules, USA).

### Statistical Analyses

Measured data were analyzed with Student's *t*-test, and the results are provided as the mean ± standard deviation (SD). Count data were analyzed with the chi-square test or Fisher's exact test. Bonferroni correction was employed to adjust false-positive results in multiple statistics. Statistical tests were mainly carried out using SPSS 19.0 (IBM, New York, USA), and *p* ≤ 0.05 was considered significant.

## Results

### Basic Characteristics

A total of 76 individuals were recruited, including 38 TLE patients and 38 healthy controls, for the present study. For stratified analysis within the TLE group, age, age at onset and disease duration were grouped according to the dichotomic classification method. The details of sex, age, age at onset, disease duration, and drug response are displayed in Table 1. No significant differences in sex or age were observed between the TLE and healthy control groups (*p* > 0.05).

**Table 1:** General characteristics of enrolled individuals.

	**TLE cases**	**Controls**	***p*-value**
Sex (male/female, *n*)	18/20	19/19	0.818
Age (mean ± SD, years old)	32.1 ± 14.4	33.4 ± 6.4	0.602
Young (<26 years old)	20.2 ± 3.3	-	-
Old (≥26 years old)	44.0 ± 10.8	-	-
Age at onset (mean ± SD, years old)	23.3 ± 16.2	-	-
Early-onset (<18 years old)	12.0 ± 5.2	-	-
Late-onset (≥18 years old)	34.6 ± 15.5	-	-
Disease duration (mean ± SD, years)	8.8 ± 9.8	-	-
Short duration (<5 years)	1.3 ± 1.5	-	-
Long duration (≥5 years)	16.3 ± 8.8	-	-
Drug response (n)	38	-	-
Sensitive patients	24	-	-
Resistant patients	14	-	-

### Bioinformatics Analysis of CpG Islands

First, 17 epilepsy-related ion channel genes were selected to assess their DNA methylation status. However, five of them, sodium voltage-gated channel alpha subunit 2 (*SCN2A*), glutamate ionotropic receptor NMDA type subunit 2D *(GRIN2D), SCN1A*, gamma-aminobutyric acid type A receptor subunit alpha1 *(GABRA1)*, and gamma-aminobutyric acid type A receptor subunit gamma2 *(GABRG2)*, were omitted from further testing because no CpG islands were found proximal to their promoters, indicating that genetic regulation of these genes is not modulated by methylation.

### DNA Methylation Levels of CpG Islands Between Patients and Controls

The DNA methylation levels of all genes tested were significantly lower in the TLE group than in the control group. After Bonferroni correction, 83.3% (10/12) of these genes, namely, gamma-aminobutyric acid type A receptor subunit beta1 *(GABRB1)*, gamma-aminobutyric acid type A receptor subunit beta2 *(GABRB2)*, gamma-aminobutyric acid type A receptor subunit beta1 *(GABRB3)*, glutamate ionotropic receptor NMDA type subunit 1 *(GRIN1)*, glutamate ionotropic receptor NMDA type subunit 2A *(GRIN2A)*, glutamate ionotropic receptor NMDA type subunit 2B *(GRIN2B), HCN1, KCNA2*, potassium voltage-gated channel subfamily B member 1 *(KCNB1)*, and potassium sodium-activated channel subfamily T member 1 *(KCNT1)*, were still different between the TLE group and the control group (Table 2). The details of the methylation of CpG islands, CpG sites and related haplotypes of these genes are shown in Table 3. Furthermore, binary logistic regression indicated that among these methylation-related genes, hypermethylated *GARAB1* was an independent risk factor for TLE (Table 4).

**Table 2:** DNA methylation status of 12 ion-channel genes between TLE cases and controls.

**Gene**	**TLE cases**	**Control**	**OR**	***p*-value**	***p*-value**
	**(Mean ± SD, %)**	**(Mean ± SD, %)**		**(*t*-test)**	**(Bonferroni)**
*GABRB1*	1.62 ± 0.52	2.39 ± 0.81	0.68	0.0000	0.0001
*GABRB2*	4.80 ± 0.96	6.28 ± 1.26	0.77	0.0000	0.0001
*GABRB3*	18.23 ± 2.14	20.29 ± 2.74	0.90	0.0005	0.0057
*GRIN1*	23.10 ± 2.96	25.62 ± 3.25	0.90	0.0007	0.0087
*GRIN2A*	2.96 ± 0.43	3.67 ± 0.76	0.81	0.0000	0.0000
*GRIN2B*	4.07 ± 0.96	5.60 ± 1.74	0.73	0.0000	0.0001
*HCN1*	4.43 ± 1.05	5.61 ± 1.42	0.79	0.0001	0.0011
*KCNA2*	1.02 ± 0.09	1.18 ± 0.13	0.87	0.0000	0.0000
*KCNB1*	21.50 ± 0.26	21.78 ± 0.35	0.99	0.0002	0.0025
*KCNQ2*	1.12 ± 0.12	1.18 ± 0.10	0.96	0.0456	0.5473
*KCNT1*	17.10 ± 1.60	19.03 ± 2.42	0.90	0.0001	0.0012
*SCN8A*	0.82 ± 0.06	0.82 ± 0.07	1.00	0.8262	9.9148

**Table 3:** Methylation details of 12 ion-channel genes between TLE cases and controls.

**Genes**	**CpG island (*n*.)**	**CpG site (** ***n*** **.)**		**Haplotype (** ***n*** **.)**	
		**All**	**Differential**	**All**	**Differential**
*GABRB1*	1	16	10	21	3
*GABRB2*	2	26	24	17	6
*GABRB3*	3	86	66	133	7
*GRIN1*	1	16	12	32	4
*GRIN2A*	3	69	37	34	4
*GRIN2B*	4	59	26	66	4
*HCN1*	2	34	22	44	4
*KCNA2*	2	28	17	24	4
*KCNB1*	3	61	19	17	0
*KCNQ2*	1	59	6	61	7
*KCNT1*	1	21	16	6	4
*SCN8A*	1	23	0	20	0

**Table 4:** Independent risk factors for DNA methylation-related ion channels evaluated by binary logistic regression.

		**B**	**S.E**,	**Wals**	**df**	**Sig**.	**Exp (B)**	**95% CI for EXP(B)**	
								**Lower**	**Upper**
Step 1^a^	Sex	0.566	0.709	0.637	1	0.425	1.761	0.439	7.064
	Age	0.049	0.035	1.979	1	0.159	1.051	0.981	1.125
	*GABRB1*	−140.522	65.451	4.61	1	0.032	9.37E−62	1.82E−117	4.83E−6
	*GABRB2*	−58.759	96.787	0.369	1	0.544	3.03E−26	1.25E−108	7.35E+56
	*GABRB3*	22.54	28.75	0.615	1	0.433	6.15E+09	2.07E−15	1.82E+34
	*GRIN1*	−2.823	15.721	0.032	1	0.858	0.059	2.47E−15	1.43E+12
	*GRIN2A*	−74.74	124.206	0.362	1	0.547	3.47E−33	6.55E−139	1.84E+73
	*GRIN2B*	−81.974	57.44	2.037	1	0.154	2.51E−36	3.21E−85	1.96E+13
	*HCN1*	106.495	67.541	2.486	1	0.115	1.78E+46	5.74E−12	5.51E+103
	*KCNA2*	−1097.276	609.439	3.242	1	0.072	0.000	0.000	1.64E+42
	*KCNB1*	−103.195	169.724	0.37	1	0.543	1.52E−45	5.17E−190	4.49E+99
	*KCNT1*	−2.542	19.018	0.018	1	0.894	0.079	5.10E−18	1.22E+15
	Constant	35.547	36.864	0.93	1	0.335	2.74E+15		

a *Variables in step 1: Sex, Age, GABRB1, GABRB2, GABRB3, GRIN1, GRIN2A, GRIN2B, HCN1, KCNA2, KCNB1, KCNT1*.

### DNA Methylation Distribution of CpG Islands Within the TLE Group

Regarding the methylation levels of the 10 differential genes identified above, further stratified analysis within the TLE group was performed, which revealed that 7 genes were downregulated in younger patients, namely, *GABRB1, GABRB2, GABRB3, GRIN1, GRIN2A, HCN1*, and *KCNB1* (Table 5). Furthermore, *GABRB1* was downregulated in TLE cases of a long duration compared to those of a short duration (Table 6). Conversely, no difference in methylation level was observed by stratified analysis of sex, age at onset and drug response (data not shown).

**Table 5:** DNA methylation of 12 ion-channel genes between young and old patients.

**Gene**	**Young**	**Old**	**OR**	***p*-value**
	**(Mean ± SD, %)**	**(Mean ± SD, %)**		
*GABRB1*	1.41 ± 0.39	1.83 ± 0.54	0.80	0.0125
*GABRB2*	4.25 ± 0.46	5.35 ± 0.99	0.80	0.0001
*GABRB3*	17.35 ± 1.67	19.11 ± 2.14	0.91	0.0093
*GRIN1*	22.12 ± 2.86	24.09 ± 2.64	0.93	0.0388
*GRIN2A*	2.78 ± 0.27	3.14 ± 0.47	0.89	0.0083
*GRIN2B*	3.87 ± 0.80	4.26 ± 1.04	0.89	0.2083
*HCN1*	4.01 ± 0.67	4.84 ± 1.17	0.84	0.0125
*KCNA2*	0.99 ± 0.07	1.05 ± 0.10	0.96	0.0592
*KCNB1*	21.40 ± 0.22	21.60 ± 0.26	0.99	0.0151
*KCNQ2*	1.13 ± 0.13	1.11 ± 0.11	1.00	0.5715
*KCNT1*	16.92 ± 1.44	17.28 ± 1.69	0.98	0.5008
*SCN8A*	0.81 ± 0.06	0.82 ± 0.05	0.96	0.5090

**Table 6:** DNA methylation of 12 ion-channel genes between short and long duration of disease.

**Gene**	**Short duration**	**Long duration**	**OR**	***p*-value**
	**(Mean ± SD, %)**	**(Mean ± SD, %)**		
*GABRB1*	1.41 ± 0.37	1.78 ± 0.56	0.80	0.0351
*GABRB2*	4.52 ± 0.85	4.89 ± 0.97	0.93	0.2026
*GABRB3*	17.62 ± 2.21	18.50 ± 1.89	0.95	0.2108
*GRIN1*	22.40 ± 3.26	23.40 ± 2.48	0.96	0.2986
*GRIN2A*	2.91 ± 0.52	2.97 ± 0.32	0.98	0.7003
*GRIN2B*	4.20 ± 0.93	3.95 ± 0.95	1.06	0.4447
*HCN1*	4.09 ± 0.95	4.63 ± 1.03	0.88	0.1219
*KCNA2*	1.00 ± 0.08	1.025 ± 0.09	0.99	0.6087
*KCNB1*	21.46 ± 0.26	21.55 ± 0.25	1.00	0.2897
*KCNQ2*	1.11 ± 0.10	1.12 ± 0.15	1.02	0.6275
*KCNT1*	17.38 ± 1.09	16.79 ± 1.85	1.04	0.2840
*SCN8A*	0.81 ± 0.06	0.82 ± 0.06	1.00	0.9887

### Protein Levels of DNA Methylation-Related Ion Channels in Peripheral Blood

**Figure 1:**
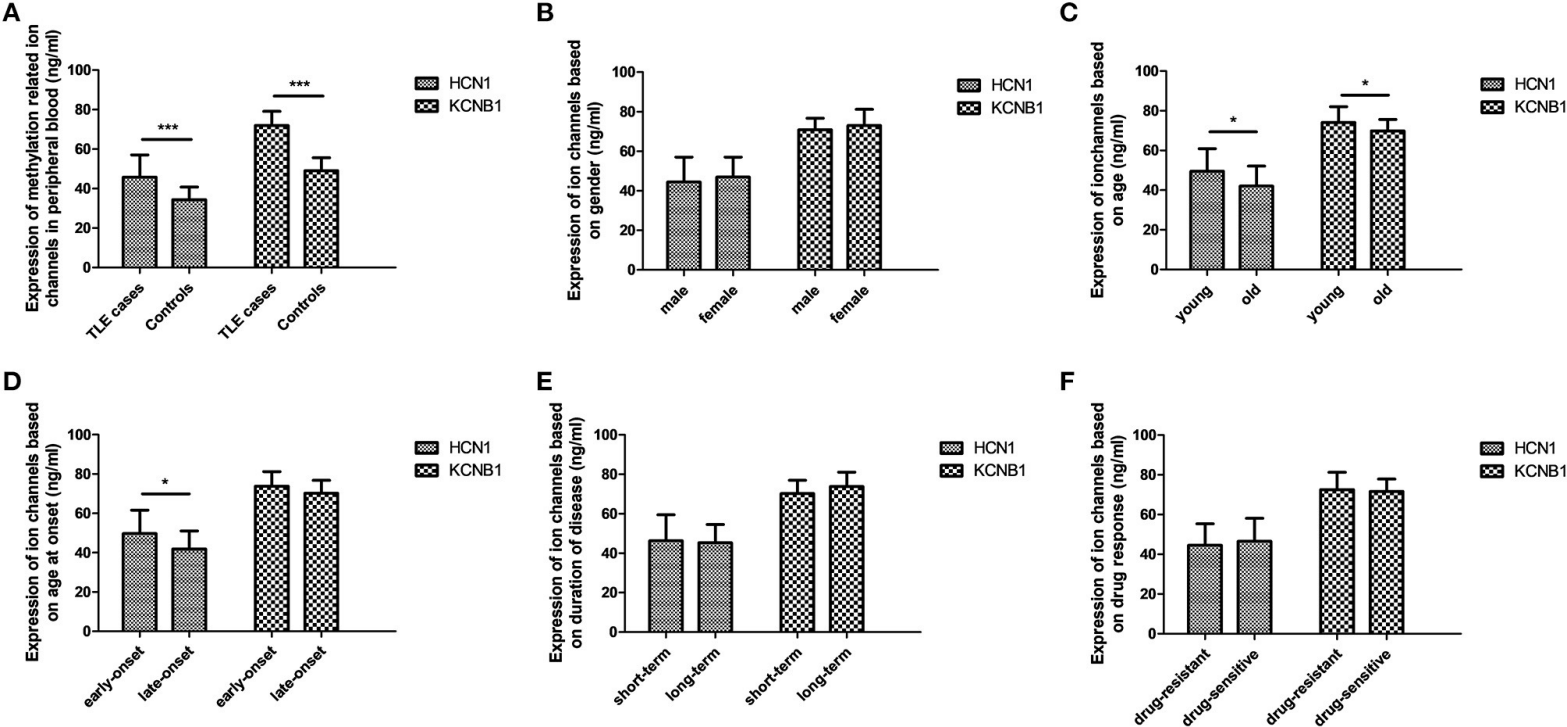
Protein levels of selected ion channel genes in peripheral blood using ELISA. The protein levels of HCN1 and KCNA2 in the TLE group reached a significant level, with a 1.3-fold change and 1.5-fold change in comparison with the control group, respectively **(A)**. Within the TLE group, the levels of HCN1 protein were significantly increased in the young patients and in the early-onset patients **(C,D)**; expression of KCNA2 was also significantly upregulated in young patients compared with old patients **(D)**, but no differences in HCN1 and KCNA2 were observed in accordance with stratified analysis of sex **(B)**, disease duration **(E)**, or drug response **(F)**. *p < 0.05; ***p < 0.001.

Although DNA methylation of most ion channel genes was significantly associated with TLE in this study, it is unknown whether DNA methylation has the potential to regulate protein levels. Hence, further experiments were performed to evaluate the effects of DNA methylation on the protein levels of selected ion-channel genes in peripheral lymphocytes; we did not use brain tissue specimens, which were hard to attain. According to ELISA, the protein levels of *HCN1* and *KCNA2* were significantly higher in the TLE group than in the control group (Figure 1).

## Discussion

In this study, the DNA methylation levels of all tested epilepsy-related ion-channel genes were significantly downregulated in the TLE group compared to the control group. Moreover, most of these genes were still different after false-positive correction. Among these identified ion channels, *HCN1* and *KCNA2* were selected to evaluate the effects of DNA methylation, and their protein levels were inversely upregulated in the TLE group compared to the control group. These findings support a key role for DNA methylation in transcriptional silencing of ion channel genes and strongly indicate that DNA methylation of ion channel genes is involved in TLE pathologies.

Nevertheless, the causal relationship between DNA methylation of ion channel genes and epileptic seizures in TLE remains unclear. Interestingly, in further stratified analysis within the TLE group, we found no significant difference in most ion channel genes between patients with long and short durations of TLE or between those with early and late onset, which indicates that recurrent seizures in TLE have no moderating effect on DNA methylation. Considering the association between DNA methylation of ion channel genes and TLE, we conclude that methylation of the ion channel genes identified in this study may be a causal factor for a predisposition toward TLE. An exception is *GABRB1*, as its methylation level was relatively low in patients with a short duration of TLE, indicating that the pathological effect of methylation of this gene might be enhanced along with a prolonged course of TLE disease.

Interestingly, during long-term observation of a healthy cohort comprising 1919 community-dwelling individuals, the incidence of epileptic seizures increased from 10.6 per 100,000 person-years between age 45 and 59, to 25.8 between age 60 and 74, to 101.1 between age 75 and 89; the cumulative incidence was 0.15% from age 45 to age 60, 0.38% to age 70, 1.01% to age 80, and 1.47% to age 90 (21), indicating an increasing risk of epileptic seizures with aging in the healthy population. In contrast, in a cohort of newly diagnosed epilepsy patients, the cumulative incidence of achieving 2-years seizure remission was 34% at 2 years, 45% at 5 years, 52% at 10 years, and 67% at 20 years (22), which indicates that the incidence of seizures declines with age in epilepsy patients. We performed stratified analysis of age in the TLE group and observed that the methylation levels of seven epilepsy-related ion channel genes, namely, *GABRB1, GABRB2, GABRB3, GRIN1, GRIN2A, HCN1*, and *KCNB1*, were relatively low in the young subgroup; this indicates that the methylation of most of the genes tested increases with age in TLE. Considering that these genes do not encode excitatory ion channels such as *GRIN1* and *GRIN2A* or inhibitory ion channels such as *GABRB3*, interaction between enhanced methylation of related genes and aging in TLE probably occurs and influences the balance of excitation and inhibition, which needs to be further investigated.

Many clinical studies support that females undergo a marginally lower incidence of epileptic seizures than males, which is usually attributed to the greater exposure of male patients to risk factors for brain injury due to symptomatic epilepsy (23). In this study, there was no significant difference in DNA methylation levels between male and female patients, indicating that methylation of epilepsy-related ion channel genes is not involved in such sex differences. In addition, we noted that nearly one-third of epilepsy patients still experienced refractory seizures, even though several new drugs have been introduced in clinical practice in recent decades (24). This study aimed to evaluate the potential of DNA methylation in drug-resistant epilepsy, but no significant difference in the ion channel genes evaluated was observed between TLE patients with drug-sensitive and drug-resistant epilepsy, indicating that methylation of epilepsy-related ion channel genes might not be involved in regulating drug resistance.

Several limitations of this research should be mentioned. First, the total number of participants was relatively small, but differential methylation of almost all of the tested ion-channel genes (10/12) between the TLE patients and controls was confirmed via Bonferroni correction; thus, the findings should be reliable to some extent. Second, most ion channels, such as *GABRB1* and *GRIN1*, play a key role in action potentials and are mainly expressed in the nervous system (25), but some channels, such as *HCN1* and *KCNA2*, also function in non-neuronal cells. Hence, further experiments were performed to evaluate the effect of differential DNA methylation on gene expression levels in peripheral blood, with the aim of indirectly confirming that the effects of DNA methylation in TLE depend on related ion channels. Third, as the genes identified as having differential methylation levels are involved in both excitatory and inhibitory ion channels, it is unclear whether their main effects on epileptic seizures are alleviation or deterioration. Nevertheless, this study observed by binary logistic regression that hypermethylated *GARAB1* was an independent risk factor for TLE, indicating that the overwhelming effect of ion channels on TLE is probably inhibitory from the perspective of DNA methylation. In addition, DNA methylation can be modified by postnatal factors and inherited by cell division by the next generation; thus, our findings might be affected by racial and environmental factors, and caution should be exercised before generalizing these findings to other populations.

## Conclusions

In this study, we systematically observed that the DNA methylation levels of 12 epilepsy-related ion channel genes were significantly downregulated in the TLE group compared to the control group. After false-positive correction, 10 genes were still differentially expressed between the groups, indicating that DNA methylation of many ion channel genes is probably involved in TLE pathologies. However, the mechanisms need to be further investigated.

## Data Availability Statement

The datasets presented in this article are not readily available because the raw genomic sequencing data is implicated in national safety. Requests to access the datasets should be directed to corresponding authors.

## Ethics Statement

The studies involving human participants were reviewed and approved by Ethics Committees of the Affiliated Hospital of Guangdong Medical University. The patients/participants provided their written informed consent to participate in this study.

## Author Contributions

HT undertook data analyses and wrote the paper. HT, ZC, JW, and JC performed the biological experiments. YC, JF, CS, HZ, and WZ collected the specimens. XZ and KL conceptualized the hypothesis and designed the study. All authors have read and approved the final manuscript.

## Conflict of Interest

The authors declare that the research was conducted in the absence of any commercial or financial relationships that could be construed as a potential conflict of interest.

## Publisher's Note

All claims expressed in this article are solely those of the authors and do not necessarily represent those of their affiliated organizations, or those of the publisher, the editors and the reviewers. Any product that may be evaluated in this article, or claim that may be made by its manufacturer, is not guaranteed or endorsed by the publisher.
